# Hsf1 Phosphorylation Generates Cell-to-Cell Variation in Hsp90 Levels and Promotes Phenotypic Plasticity

**DOI:** 10.1016/j.celrep.2018.02.083

**Published:** 2018-03-20

**Authors:** Xu Zheng, Ali Beyzavi, Joanna Krakowiak, Nikit Patel, Ahmad S. Khalil, David Pincus

**Affiliations:** 1 Whitehead Institute for Biomedical Research, Cambridge, MA, USA; 2 Department of Mechanical Engineering, Boston University, Boston, MA, USA; 3 Department of Biomedical Engineering and Biological Design Center, Boston University, Boston, MA, USA; 4 Wyss Institute for Biologically Inspired Engineering, Harvard University, Boston, MA, USA

## Abstract

Clonal populations of cells exhibit cell-to-cell variation in the transcription of individual genes. In addition to this noise in gene expression, heterogeneity in the proteome and the proteostasis network expands the phenotypic diversity of a population. Heat shock factor 1 (Hsf1) regulates chaperone gene expression, thereby coupling transcriptional noise to proteostasis. Here we show that cell-to-cell variation in Hsf1 activity is an important determinant of phenotypic plasticity. Budding yeast cells with high Hsf1 activity were enriched for the ability to acquire resistance to an antifungal drug, and this enrichment depended on Hsp90, a known phenotypic capacitor and canonical Hsf1 target. We show that Hsf1 phosphorylation promotes cell-to-cell variation, and this variation, rather than absolute Hsf1 activity, promotes antifungal resistance. We propose that Hsf1 phosphorylation enables differential tuning of the proteostasis network in individual cells, allowing populations to access a range of phenotypic states.

## In Brief

Zheng et al. reveal that the master transcriptional regulator of proteostasis, Hsf1, generates cell-to-cell variation in the expression of Hsp90 and other chaperones. This variation is driven by differential Hsf1 phosphorylation and results in the ability of yeast cells to acquire antifungal resistance, a hallmark of phenotypic plasticity.

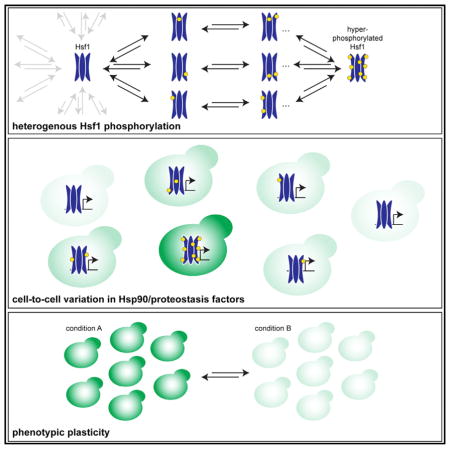

## INTRODUCTION

Genetically identical cells grown together in the same environment nonetheless display cell-to-cell variation in gene expression (Colman-Lerner et al., 2005; Elowitz et al., 2002; Raser and O’Shea, 2004, 2005; Weinberger et al., 2005). While most frequently observed in microorganisms, such as bacteria and yeast, gene expression variation is also found in developing mammalian cells and human embryonic stem cells (Silva and Smith, 2008; Stelzer et al., 2015). Such variation has been proposed to be the mechanistic underpinning of lineage commitment during human development, the epithelial-to-mesenchymal transition in cancer metastasis, organ regeneration in planarians, bacterial persistence in the presence of antibiotics, and the ability of yeast cells to remain fit in fluctuating environments (Harms et al., 2016; Newman et al., 2006; Oderberg et al., 2017; Silva and Smith, 2008; Ye and Weinberg, 2015). Although differences in cell size, cell-cycle position, and chromatin state can partially account for cell-to-cell variation, much of the variability has been attributed to the inherently stochastic process of gene expression (Colman-Lerner et al., 2005; Raj and van Oudenaarden, 2008; Raser and O’Shea, 2005). Despite the underlying stochasticity, gene expression varies widely across the genome, with some sets of genes showing very low variation among cells (e.g., ribosomal protein genes) and other sets of genes (e.g., stress-responsive genes) showing high levels of variation (Newman et al., 2006). Yet individual genes within these regulons show strong covariance, indicating the source of the variation lies in the activity of upstream transcription factors and signaling pathways (Stewart-Ornstein et al., 2012). As such, cell-to-cell variation may be a property that is under genetic control and can be tuned up and down over evolution.

On top of this gene expression variation, cell-to-cell differences exist in the state of the proteome. Perhaps the most striking examples of proteome variation come from prion proteins, which can exist in either soluble or self-templating amyloid conformations (Shorter and Lindquist, 2005). Prions have been shown to have the ability to broadly reshape the proteome by challenging chaperones and other components of the protein homeostasis (proteostasis) machinery and even by globally altering protein translation (Serio and Lindquist, 1999; Shorter and Lindquist, 2008). Moreover, chaperones can exist in large heterotypic complexes that differ among cells in what has been termed the epichaperome, giving rise to altered susceptibility of cancer cells to drugs that target the essential chaperone heat shock protein (Hsp) 90 (Rodina et al., 2016). By buffering the proteome and stabilizing near-native protein folds, Hsp90 has been shown to mask latent genetic variation in fruit flies and plants and to enhance the ability of yeast cells to acquire novel phenotypes, such as resistance to antifungal drugs (Cowen and Lindquist, 2005; Queitsch et al., 2002; Rutherford and Lindquist, 1998). In this regard, Hsp90 has been termed a phenotypic capacitor (Sangster et al., 2004).

Heat shock factor 1 (Hsf1) regulates the expression of many components of the proteostasis machinery, including Hsp90, in eukaryotes from yeast to humans (Anckar and Sistonen, 2011). In unstressed budding yeast cells, a different chaperone, Hsp70, binds to Hsf1 and restrains its activity. Upon heat shock, Hsp70 dissociates from Hsf1, leaving Hsf1 free to induce expression of its target genes (Zheng et al., 2016). Heat shock also triggers Hsf1 hyperphosphorylation. Although phosphorylation is a conserved hallmark of Hsf1 activation, it is dispensable for acute Hsf1 activity during heat shock in both yeast and human cells (Budzyński et al., 2015; Zheng et al., 2016). Rather than switching Hsf1 on, phosphorylation enables Hsf1 to sustain increased activity during prolonged exposure to elevated temperature (Zheng et al., 2016). Here we identify a novel role for Hsf1, and Hsf1 phosphorylation, that may have provided a strong selective advantage during evolution. We show that Hsf1 generates cell-to-cell variation in Hsp90 levels, which in turn contributes to the ability of *Saccharomyces cerevisiae* to acquire resistance to the antifungal drug fluconazole. We find that the ability of Hsf1 to become phosphorylated is a key factor in generating population-level heterogeneity in its activity. We propose that by coordinately controlling cytosolic chaperone genes, including Hsp90, Hsf1 promotes phenotypic plasticity.

## RESULTS

### Differential Cell-to-Cell Variation in Hsf1 Activity in Response to Heat Shock and AZC

**Figure 1:**
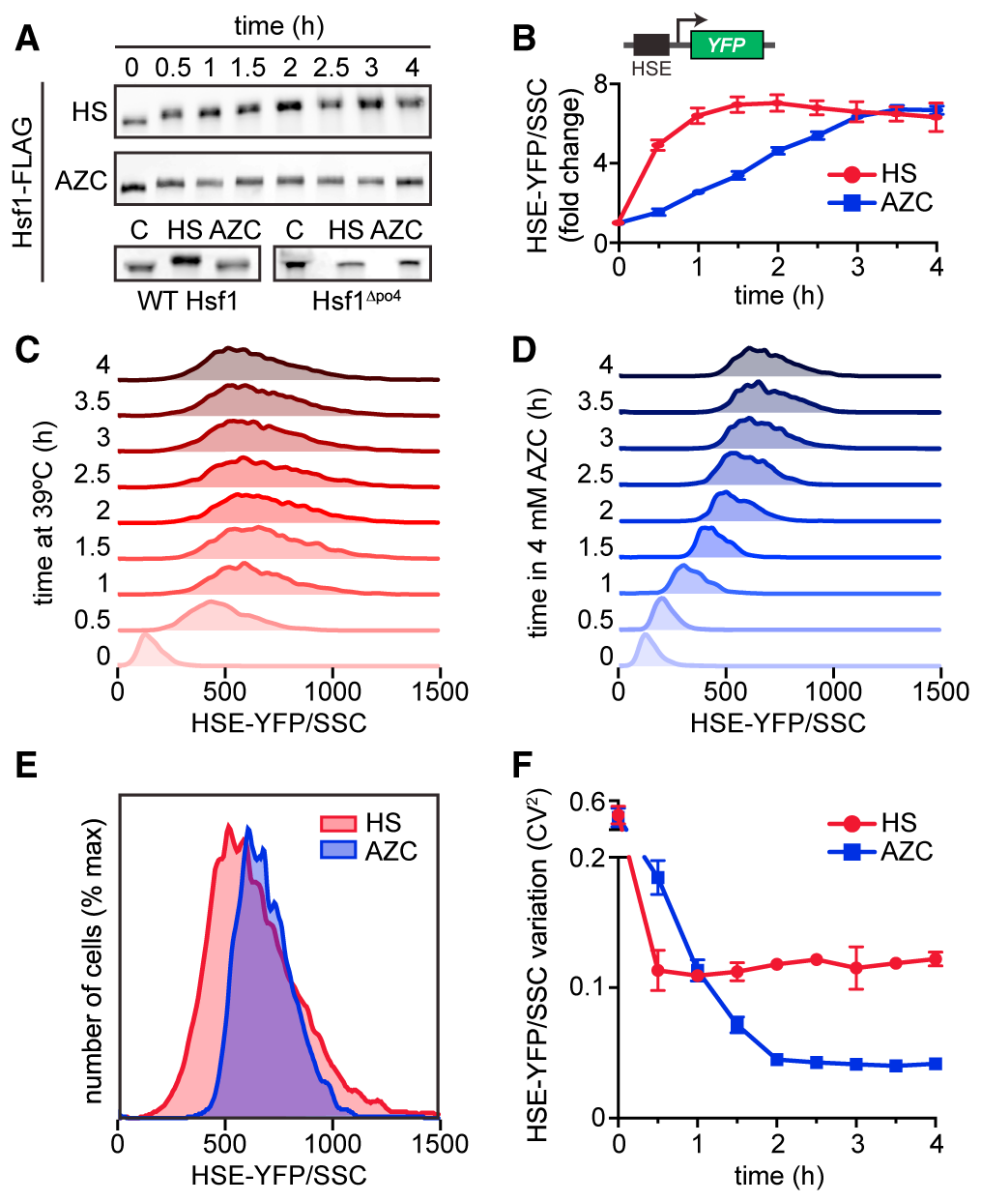
Differential Cell-to-Cell Variation in Hsf1 Activity in Response to Heat Shock and AZC (A) Anti-FLAG western blot showing Hsf1 phosphorylation by electrophoretic mobility shifts over time in response to heat shock at 39°C or 4 mM AZC. Lower panel shows Hsf1 mobility under control conditions (C) and in response to heat shock at 39°C (HS) and 4 mM AZC for 4 hr. (B) Hsf1 activity quantified by the levels of the HSE-YFP transcriptional reporter measured by flow cytometry over time in response to heat shock at 39°C or 4 mM AZC. The reporter consists of four repeats of the heat shock element (HSE) recognized by Hsf1 upstream of a crippled *CYC1* promoter. For each cell, the YFP level was normalized by side scatter (HSE-YFP/SSC) to control for cell size. Each point represents the median of the fluorescence distribution of 10,000 cells averaged over three biological replicates; error bars show the SD of the replicates. (C) Single-cell fluorescence distributions over a 4-hr heat shock time course. For each cell, the YFP level was normalized by side scatter (HSE-YFP/side scatter) to control for cell size. (D) As in (C), but over a time course of treatment with 4 mM AZC. (E) Overlay of the 4-hr time points from (C) and (D). (F) Quantification of cell-to-cell variation in HSE-YFP/side scatter levels over time courses in (C) and (D) using the square of the coefficient of variation (CV^2^) as a metric.

In addition to heat shock, Hsf1 is known to respond to a variety of chemical stressors that impair proteostasis. In particular, the small molecule azetidine 2-carboxylic acid (AZC) is known to strongly activate Hsf1 (Trotter et al., 2002). AZC is a proline analog that is charged onto tRNA^pro^ and incorporated into nascent proteins during translation, impairing their subsequent folding (Fowden and Richmond, 1963). Our previous mass spectrometry data indicated that Hsf1 displays distinct phosphorylation patterns in cells that had been heat shocked compared to cells treated with AZC (Zheng et al., 2016). To explore these differences, we monitored phosphorylation of FLAG-tagged Hsf1 following heat shock and treatment with AZC by observing its electrophoretic mobility over time by western blot. Heat shock induced progressive and sustained Hsf1 phosphorylation, leading to a dramatic shift in its mobility over the heat shock time course (Figure 1A). By contrast, AZC led to only a modest shift in Hsf1 mobility (Figure 1A). Despite these differences in phosphorylation, both heat shock and AZC robustly induced Hsf1 transcriptional activity, as measured by flow cytometry of cells expressing heat shock element (HSE)-yellow fluorescent protein (YFP), a fluorescent reporter of Hsf1 transcriptional activity (Figure 1B) (Zheng et al., 2016). While heat shock resulted in rapid HSE-YFP induction, plateauing after 1 hr, AZC induced Hsf1 activity with delayed but sustained kinetics, leading to the same maximal output level as heat shock after 4 hr (Figure 1B).

Along with altered activation kinetics, we observed differences in the single-cell fluorescence distributions: the populations of cells that had been heat shocked showed a broad distribution of HSE-YFP levels, while cells treated with AZC showed narrower distributions, indicating reduced levels of cell-to-cell variation in Hsf1 activity. To account for potential differences in cell size, we normalized each cell’s HSE-YFP fluorescence by its size, as measured by side scatter (SSC), and plotted the resulting normalized distributions over the heat shock and AZC time courses (Figures 1C and 1D) (Stewart-Ornstein et al., 2012). Direct comparison of the HSE-YFP/side scatter distributions following 4 hr of heat shock or treatment with AZC reveals that despite a slightly higher average level of Hsf1 activation in the AZC-treated cells, cellular heterogeneity is greatly reduced compared to heat-shocked cells (Figure 1E). Using Levene’s test, we determined that these distributions have significantly different variances (Table S1). To quantify this cell-to-cell variation in the HSE-YFP reporter, we calculated the square of the coefficient of variation (CV^2^) (Stewart-Ornstein et al., 2012). While the CV^2^ drops as the mean increases for both heat-shocked and AZC-treated cells, it is 3-fold higher in heat-shocked cells by the end of the experiment (Figure 1F). These data show that although heat shock and AZC both potently activate Hsf1, they do so with distinctive features. Heat shock triggers rapid Hsf1 activation coupled to high levels of phosphorylation and results in a high degree of cell-to-cell variation in the HSE-YFP reporter. By contrast, AZC induces slow, sustained Hsf1 activation; modest Hsf1 phosphorylation; and reduced CV^2^ in the HSE-YFP reporter.

### Hsf1 Phosphorylation Generates Cell-to-Cell Variation during Heat Shock

**Figure 2:**
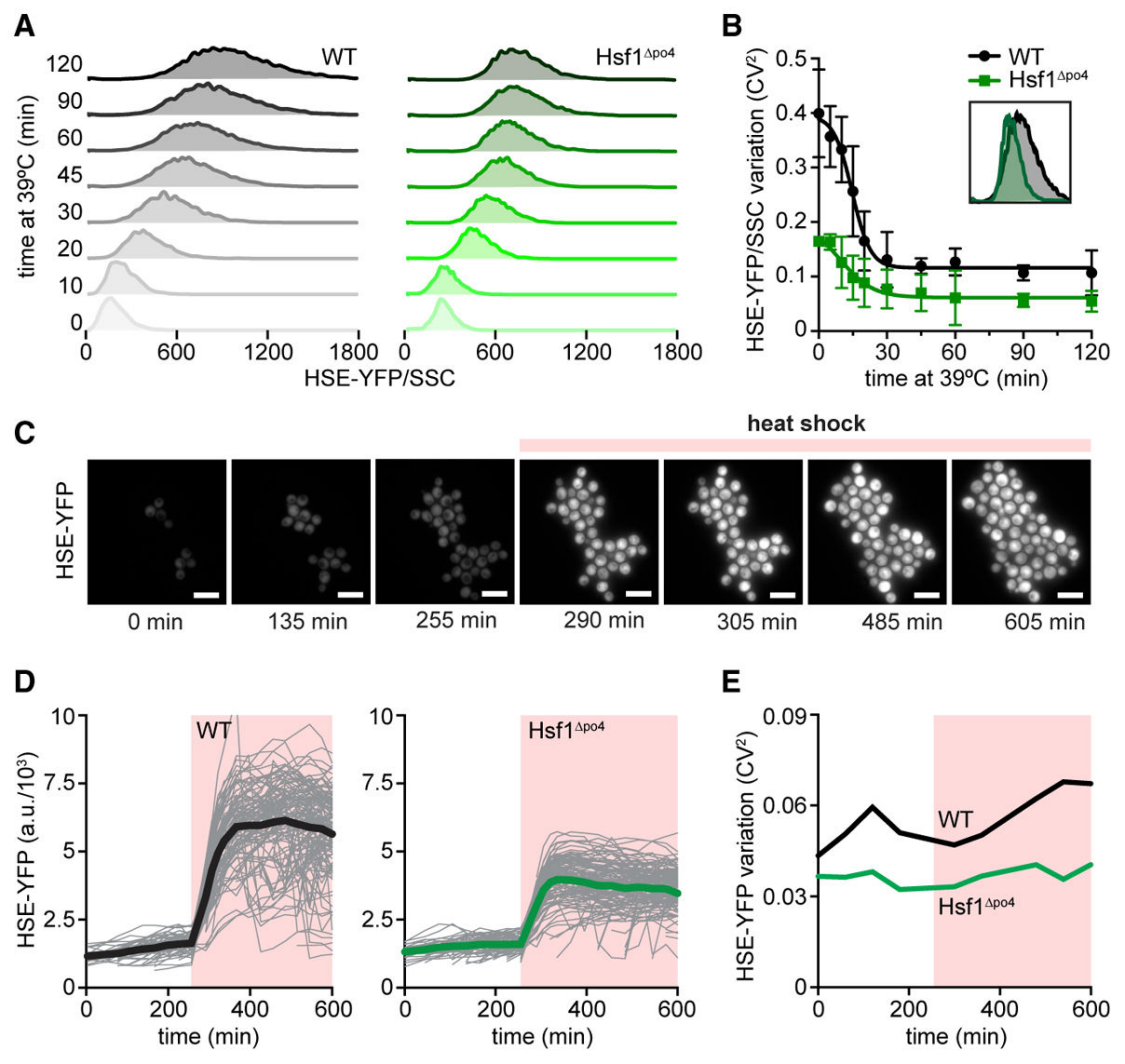
Hsf1 Phosphorylation Generates Cell-to-Cell Variation during Heat Shock (A) Single-cell fluorescence distributions over a heat shock time course in wild-type cells and cells expressing Hsf1^Δpo4^ as the only copy of Hsf1. For each cell, the YFP level was normalized by side scatter (HSE-YFP/side scatter) to control for cell size. (B) Quantification of cell-to-cell variation in HSE-YFP/side scatter levels over time courses in (A). Error bars represent the SD of three biological replicates. Inset shows an overlay of the distributions of 4-hr time points in (A). (C) Images of wild-type cells expressing the HSE-YFP reporter growing over time in a microfluidic device at 25°C and shifted to 39°C at the indicated time. Scale bar, 10 μm. (D) Quantification of HSE-YFP levels over time in the microfluidic device in wild-type cells and cells expressing Hsf1^Δpo4^ as the only copy of Hsf1. For each cell, the total YFP fluorescence was divided by cell area to account for cell-size differences. Each gray trace represents the trajectory of a single cell, and thick lines show population averages. (E) Quantification of cell-to-cell variation in HSE-YFP levels over time courses in (D).

Because increased Hsf1 phosphorylation was associated with increased cell-to-cell variation in the HSE-YFP reporter, we wondered whether Hsf1 phosphorylation was responsible for increasing noise during heat shock. To test this, we leveraged a non-phosphorylatable mutant we had previously generated, Hsf1^Δpo4^, in which we mutated all 152 possible sites of phosphorylation to alanine (Zheng et al., 2016). In contrast to wild-type Hsf1, Hsf1^Δpo4^ does not shift in its mobility in response to heat shock or AZC treatment (Figure 1A). We performed a heat shock time course and measured the HSE-YFP reporter by flow cytometry in wild-type cells and cells expressing Hsf1^Δpo4^. After normalizing for cell size, we observed reduced noise in the HSE-YFP reporter in the Hsf1^Δpo4^ cells compared to wild-type throughout the heat shock time course (Figures 2A and 2B). As an orthogonal approach to measure cell-to-cell variation in Hsf1 activity, we developed a microfluidic-based assay to measure the HSE-YFP reporter in single cells over time in response to heat shock using a novel platform that enables precision temperature control (Figure 2C; Figure S1). In agreement with the flow cytometry data, cells expressing Hsf1^Δpo4^ showed lower noise in HSE-YFP levels over the heat shock time course (Figures 2D and 2E). Thus, Hsf1 phosphorylation promotes cell-to-cell variation in Hsf1 activity during heat shock.

In addition to removing all sites of phosphorylation, we employed a mutant, Hsf1^PO4^* in which we mimicked constitutive phosphorylation by making 116 aspartate substitutions (Zheng et al., 2016). This mutant showed dramatically increased Hsf1 activity, as measured by the HSE-YFP reporter, as well as reduced cell-to-cell variation (Figures S2A–S2D). However, most of the decrease in CV^2^ can be attributed to the increase in activity, making this mutant difficult to interpret. Nonetheless, fixing Hsf1 in a state mimicking constitutive phosphorylation reduced cell-to-cell variation, consistent with the notion that variation in Hsf1 phosphorylation underlies variation in Hsf1 activity.

### Hsp90 Expression Displays Hsf1-Dependent Cell-to-Cell Variation

**Figure 3:**
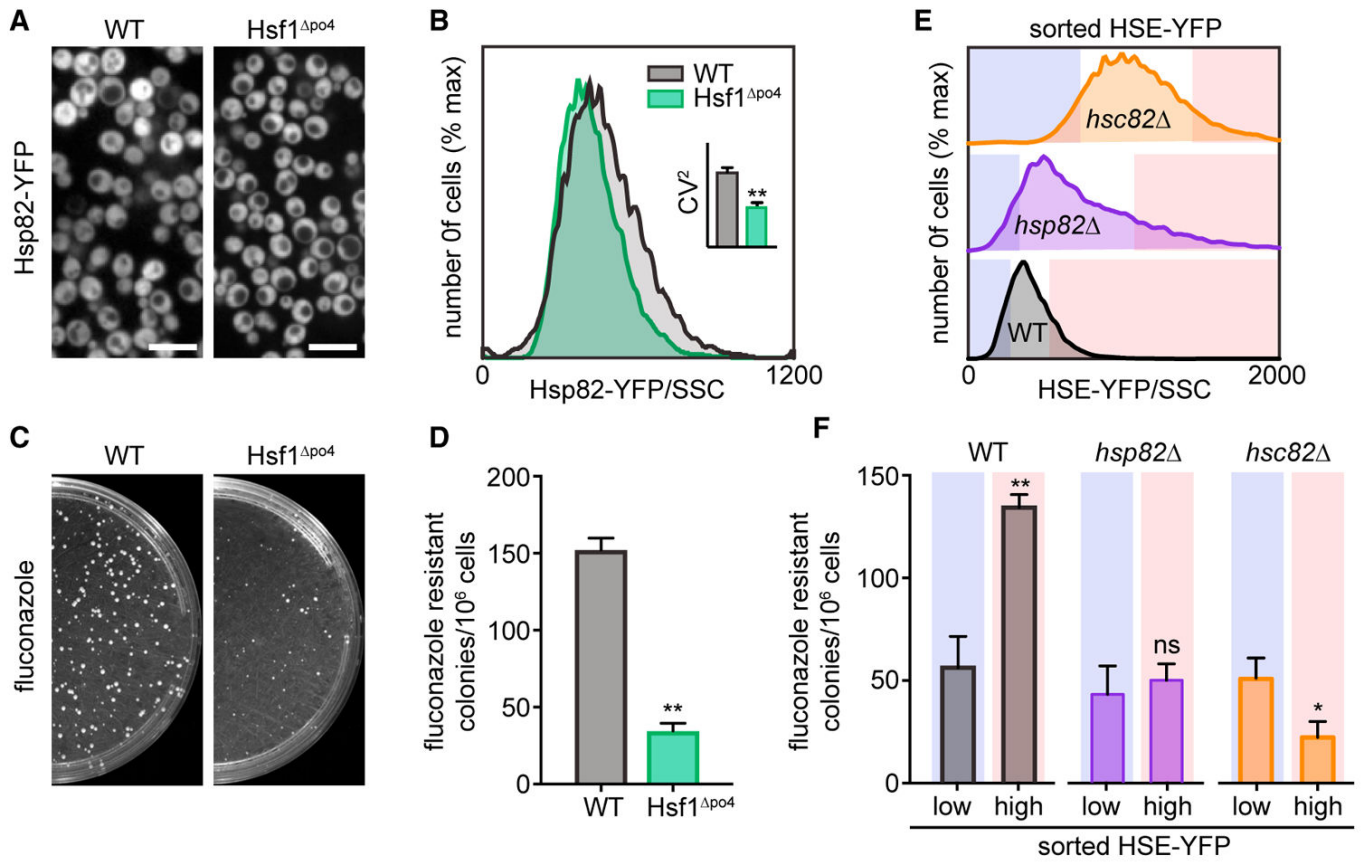
Cell-To-Cell Variation in Hsf1 Activity Promotes Antifungal Resistance in an Hsp90-Dependent Manner (A) Spinning disc confocal images of wild-type cells and Hsf1^Δpo4^ cells expressing Hsp82-YFP. Scale bar, 10 μm. (B) Single-cell fluorescence distributions of wild-type cells and Hsf1^Δpo4^ cells expressing Hsp82-YFP, as determined by flow cytometry. For each cell, the YFP level was normalized by side scatter (Hsp82-YFP/side scatter) to control for cell size. The cells in the top 10% of the distribution have on average ~2.3-fold more Hsp82 than the median of the full distribution. (C) Appearance of fluconazole-resistant colonies in wild-type cells and Hsf1^Δpo4^ cells. 10^6^ cells were plated on YPD plates supplemented with 128 μg/mL of fluconazole and incubated at room temperature for 4 days. (D) Quantification of the number of fluconazole-resistant colonies from three biological replicates of the experiment shown in (C). Error bars show the SD of the replicates (*p < 0.05; **p < 0.01 by two-tailed t test). (E) Single-cell fluorescence distributions of HSE-YFP/side scatter in wild-type and Hsp90 mutant cells under basal conditions. Deletion of *HSP82* leads to a modest increase in HSE-YFP levels, while deletion of *HSC82* leads to a pronounced increase in HSE-YFP levels. The blue and red boxes indicate the tails of the distribution with the highest- and lowest-expressing 10% of cells, respectively. (F) Quantification of the number of fluconazole-resistant colonies in the tails of the distributions shown in (E) (*p < 0.05; **p < 0.01 by two-tailed t test).

To investigate functional roles for cell-to-cell variation in Hsf1 activity, we monitored expression of Hsp90, an endogenous Hsf1 target gene known to influence phenotypic plasticity (Cowen and Lindquist, 2005). Budding yeast encodes two Hsp90 paralogs, Hsc82 and Hsp82, with Hsc82 being highly expressed under all conditions and Hsp82 showing both basal and inducible expression in response to heat shock (Solís et al., 2016). We tagged Hsp82 with YFP and monitored its expression in single cells expressing either wild-type Hsf1 or Hsf1^Δpo4^ by fluorescence microscopy and flow cytometry. Consistent with the HSE-YFP reporter, Hsp82-YFP expression showed greater variability among wild-type cells than Hsf1^Δpo4^ cells (Figures 3A and 3B), though this did not quite reach significance by Levene’s test (p = 0.08) (Table S1). However, the CV^2^ was significantly lower for the Hsf1^Δpo4^ cells compared to wild-type (Figure 3B, inset).

Prior studies have implicated Hsp90 as a phenotypic capacitor that promotes the ability of cells to acquire novel phenotypes (Sangster et al., 2004). In particular, in fungi, Hsp90 has been shown to increase the rate at which cells acquire resistance to the antifungal drug fluconazole (Cowen and Lindquist, 2005). We hypothesized that the increased cell-to-cell variation in Hsp82-YFP expression levels in wild-type cells compared to Hsf1^Δpo4^ cells would translate into increased fluconazole resistance. Wild-type cells generated significantly more fluconazole-resistant colonies than Hsf1^Δpo4^ cells or Hsf1^PO4^* cells (Figures 3C and 3D; Figures S2E and S2F).

### Acquired Fluconazole Resistance Is Enriched in Cells with High Hsf1 Activity

Because variation in Hsp82-YFP levels and the HSE-YFP reporter correlated with phenotypic plasticity, we wondered whether individual cells with elevated HSE-YFP levels would be more likely to become resistant to fluconazole. To test this, we used fluorescence-activated cell sorting (FACS) to isolate the tails of the HSE-YFP distribution—the 10% of cells with the highest and lowest HSE-YFP expression levels—in wild-type cells, *hsp82*Δ cells, and *hsc82*Δ cells (Figure 3E). In wild-type cells, the tail with high HSE-YFP expression was enriched for fluconazole-resistant colonies over the low-expression tail (Figure 3F). By contrast, the high-expression tails of the *hsp82*Δ and *hsc82*Δ cells showed no enrichment relative to the low-expression tails, with the *hsc82*Δ high-expression tail showing decreased resistance (Figure 3F). These results indicate that cells with higher levels of Hsf1 activity have an increased ability to acquire resistance to an antifungal drug, and this ability depends on Hsp90.

### Fluconazole Resistance Correlates with Variation in Hsf1 Activity, Not Its Magnitude

**Figure 4:**
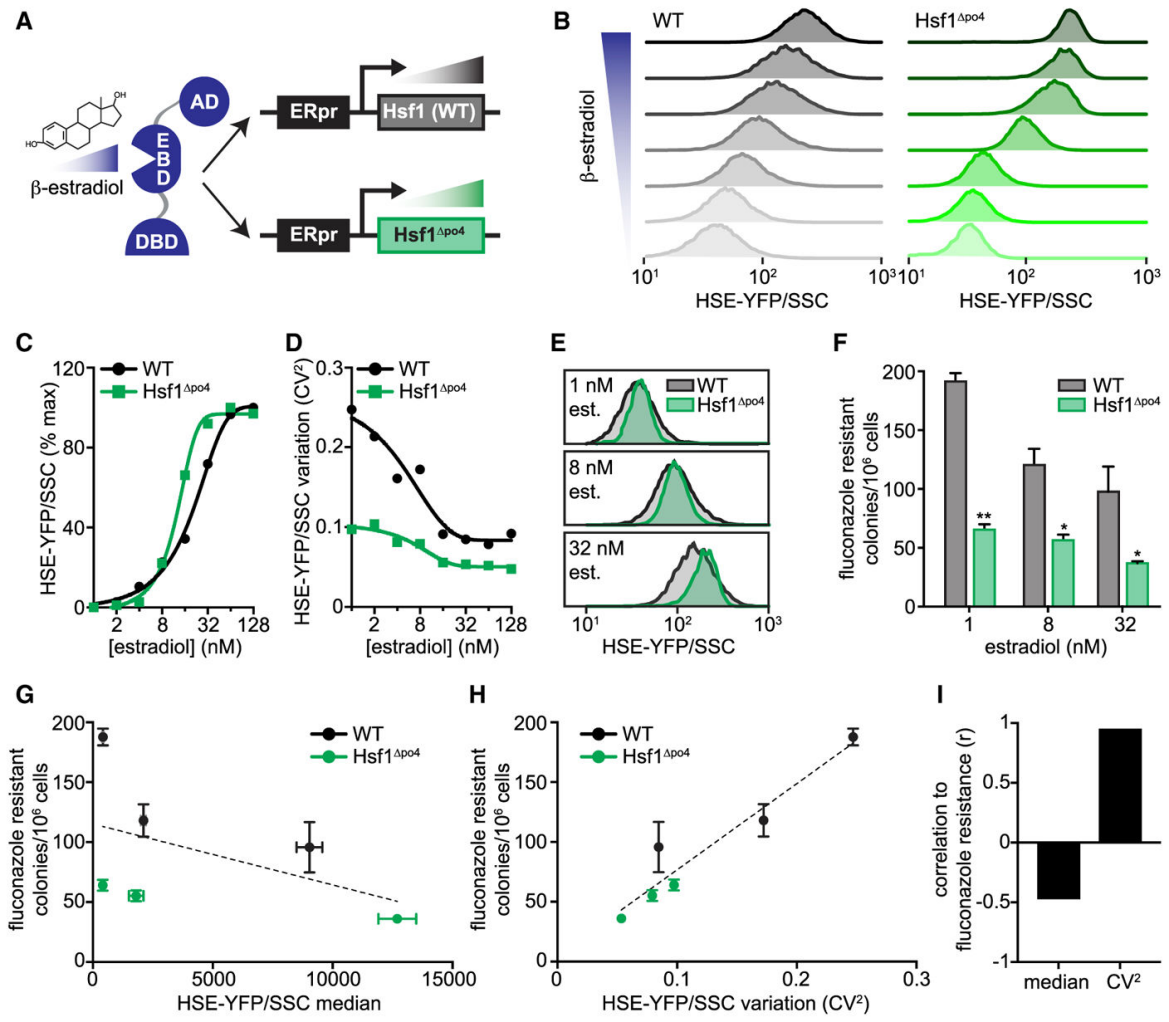
Fluconazole Resistance Correlates with Cell-to-Cell Variation in Hsf1 Activity (A) Schematic showing the estradiol-inducible system used to titrate expression of wild-type Hsf1 and Hsf1^Δpo4^. A chimeric transcription factor containing the ligand binding domain of the human estrogen receptor drives the expression of Hsf1 as a function of the concentration of estradiol added to the growth media. (B) Single-cell HSE-YFP/side scatter fluorescence distributions in cells treated across a dose response of estradiol, ranging from 2 to 128 nM in cells expressing wild-type Hsf1 (gray) or Hsf1^Δpo4^ (green) as the only copy of Hsf1. Cells were incubated in log phase for 18 hr in the presence of the indicated dose of estradiol to achieve steady state before measuring. (C) Quantification of the median HSE-YFP/side scatter across the estradiol dose response in wild-type and Hsf1^Δpo4^ cells relative to the maximum value. (D) Quantification of the cell-to-cell variation (CV^2^) of the HSE-YFP/side scatter distributions across the estradiol dose response in wild-type and Hsf1^Δpo4^ cells. (E) Overlays of the HSE-YFP/side scatter distributions from wild-type and Hsf1^Δpo4^ cells treated with the indicated concentrations of estradiol. (F) Quantification of fluconazole-resistant colonies in wild-type and Hsf1^Δpo4^ cells in the presence of the indicated doses of estradiol (*p < 0.05; **p < 0.01 by two-tailed t test). (G) Fluconazole-resistant wild-type and Hsf1^Δpo4^ colonies plotted as a function of median Hsf1 activity as measured by the HSE-YFP reporter. Dashed line is a linear regression to all six data points showing a negative correlation. (H) Fluconazole-resistant wild-type and Hsf1^Δpo4^ colonies plotted as a function of the variation in Hsf1 activity as measured by the CV^2^ of the HSE-YFP reporter. Dashed line is a linear regression to all six data points showing a positive correlation. (I) Correlation coefficients (r) of the data plotted in (G) and (H).

Because the high-expression tail of the HSE-YFP distribution was enriched for cells able to acquire resistance to fluconazole, we hypothesized that if we could increase the average HSE-YFP expression, we could increase the ability of cells to acquire fluconazole resistance. To test this, we expressed wild-type Hsf1 from a promoter under the control of a synthetic, estradiol-responsive (ER) transcription factor (Zheng et al., 2016). In this way, we could titrate the amount of estradiol to tune the expression level of Hsf1 and thereby control the expression level of its target genes and the HSE-YFP reporter. In parallel, we expressed Hsf1^Δpo4^ using the same system to determine whether the deficit in cell-to-cell variation could be overcome by increasing the absolute expression (Figure 4A). We grew the wild-type and Hsf1^Δpo4^ cells in a dilution series of estradiol, maintained growth at optical density 600 (OD_600_) < 0.5 for 18 hr, and measured the HSE-YFP reporter. Estradiol caused dose-dependent induction of the HSE-YFP in both wild-type and Hsf1^Δpo4^ cells, though with a steeper curve for Hsf1^Δpo4^ cells (Figures 4B and 4C). At all doses of estradiol, wild-type cells had higher CV^2^ in the HSE-YFP reporter than Hsf1^Δpo4^ cells (Figures 4B and 4D). We tested the ability of cells expressing wild-type Hsf1 and Hsf1^Δpo4^ at low (1 nM estradiol), medium (8 nM estradiol), and high (32 nM estradiol) levels to acquire resistance to fluconazole (Figure 4E). Surprisingly, increasing median HSE-YFP did not increase resistance: both wild-type and Hsf1^Δpo4^ cells showed maximal resistance at low Hsf1 expression (Figure 4F). However, wild-type cells showed greater resistance at all Hsf1 expression levels than did Hsf1^Δpo4^ cells (Figure 4F). Thus, increased variation in HSE-YFP levels, not increased median levels, positively correlates with fluconazole resistance (Figures 4G–4I).

Finally, to test the relationships among Hsf1 activity, cell-to-cell variation, and fluconazole resistance in a more natural manner, we monitored fluconazole resistance in wild-type cells that had been pre-treated with AZC, heat shocked, or left untreated. We found that untreated cells formed the most fluconazole-resistant colonies, followed by heat-shocked cells, and that AZC-treated cells formed the fewest resistant colonies (Figure S3A). Consistent with the synthetic estradiol-mediated induction experiments, this rank order positively correlates with cell-to-cell variation in Hsf1 activity, not with absolute Hsf1 activity (Figures S3B and S3C).

## DISCUSSION

Cell-to-cell variation in gene expression and the state of the proteome have been proposed to contribute to adaptability in populations of genetically identical cells. Here we identify a single regulatory mechanism that couples variation at the transcriptional and proteomic levels: phosphorylation of the transcription factor Hsf1. We found that when wild-type Hsf1 is activated and concomitantly hyperphosphorylated, as is the case during heat shock, cells display high variation in their expression of downstream Hsf1 target genes. By contrast, when Hsf1 is activated without hyperphosphorylation following treatment with AZC, cells show more uniform target gene expression. We attribute the differential phosphorylation to more signaling pathways responding to heat shock than to AZC. Principally, we hypothesize that heat shock-dependent inactivation of the Ras/protein kinase A (Ras/PKA) pathway is likely to de-repress a number of kinase pathways that may lead to hyperphosphorylation of Hsf1. The state of the Ras/PKA pathway is thought to be a major source of cell-to-cell variation (Stewart-Ornstein et al., 2012).

To test whether Hsf1 phosphorylation is linked to cell-to-cell variation, we employed a mutant we had previously generated that lacks the ability to be phosphorylated. Removing the ability of Hsf1 to be phosphorylated reduced cell-to-cell variation, indicating that much of the variation depends on the ability of Hsf1 to be phosphorylated. The remaining variation is likely to arise from intrinsic sources such as chromatin state, cell-cycle position, and metabolic state (Eldar and Elowitz, 2010). Because Hsf1 has so many possible sites of phosphorylation (≥73), we find it likely that the ensemble of differentially phosphorylated molecules underlies the variation, rather than a single phosphorylation site. Because the Hsf1 regulon consists of chaperones and other proteostasis factors, variation in Hsf1 activity is likely to lead to variation in the proteostasis network and the state of the proteome.

Functionally, we demonstrated that cell-to-cell variation in Hsf1 activity is correlated with the ability of cells to acquire resistance to the antifungal drug fluconazole, largely via expression of the known phenotypic capacitor Hsp90. While wild-type cells with high levels of Hsf1 activity were enriched for the ability to acquire fluconazole resistance, cells with reduced levels of Hsp90 lost this enrichment. This observation suggested that cells with more Hsf1 activity would have more Hsp90 and therefore show increased antifungal resistance. However, synthetically increasing Hsf1 activity resulted in diminished fluconazole resistance. Moreover, increased expression of non-phosphorylatable Hsf1^Δpo4^, which showed reduced cell-to-cell variation in its activity compared to wild-type Hsf1, could not compensate for its reduced ability to support fluconazole resistance. Thus, antifungal resistance correlates with the variation in Hsf1 activity, rather than with the magnitude of Hsf1 activity.

Unstressed wild-type cells show the highest level of cell-to-cell variation in Hsf1 activity, and unstressed cells show the greatest ability to acquire fluconazole resistance—greater even than that of cells that had been heat shocked to trigger Hsf1 phosphorylation (Figure S3). This variation in unstressed cells still depends on Hsf1 phosphorylation, because cells expressing Hsf1^Δpo4^ show reduced variation (Figure 2). Thus, it is not hyperphosphorylation of Hsf1 that drives resistance to fluconazole; it is the ability of Hsf1 to be differentially phosphorylated in different cells that underlies much of the heterogeneity in its activity. Hsf1 is not “off” in unstressed cells; it is poised at an intermediate level of activity. By using CV^2^ as a metric for cell-to-cell variation, which normalizes the SD by the mean, we retain relevant cell-to-cell variation under basal conditions. In unstressed cells, there is no external activating signal for Hsf1 that synchronizes the population, so intrinsic states in each cell are likely to dominate the signals controlling Hsf1 activity and phosphorylation.

How does Hsf1 phosphorylation lead to cell-to-cell variation, and why does variation promote fluconazole resistance? It is likely that differential activation of cell-cycle, metabolic, and stress-responsive kinase pathways results in differential Hsf1 phosphorylation states and leads to distinct levels of transcriptional activity in single cells. However, the finding that variation in Hsf1 activity, rather than its absolute activity, drives resistance is more intriguing. Although cells with elevated Hsf1 activity produce more Hsp90, they also overproduce the rest of the Hsf1 regulon, which may be maladaptive. In addition, with forced Hsf1 activity, cells are unable to dynamically regulate Hsf1 to precisely tune its activity according to need. While a high level of Hsf1 activity, and thus Hsp90 levels, may be beneficial early in the process of acquiring resistance to fluconazole, it may be that a subsequent reduction in Hsf1 activity promotes proliferation. The capacity for dynamic and coordinated control over the proteostasis network may endow cells with the plasticity required to maintain fitness in fluctuating environments. By enabling both population-level variation and dynamic control over the proteostasis network, Hsf1 is a powerful phenotypic capacitor.

## EXPERIMENTAL PROCEDURES

### Yeast Strains and Cell Growth

Yeast cells were cultured in synthetic complete media with dextrose (SDC) media as described (Zheng et al., 2016). Strain information and genotypes are detailed in Table S2.

### Flow Cytometry

Flow cytometry was performed as described (Zheng et al., 2016). Data were processed in FlowJo 10. Data were left ungated, and YFP fluorescence was normalized by side scatter for each cell.

### Microfluidic Heat Shock Time Courses

Experimental details are described in Supplemental Experimental Procedures.

### Spinning Disc Confocal Imaging

Imaging was performed as described (Zheng et al., 2016).

### Fluconazole-Acquired Resistance Assay

Wild-type and Hsf1^Δpo4^ cells were grown to mid-log phase, and 10^6^ cells were plated on YPD plates with 128 μg/mL fluconazole + 25 nM estradiol. Plates were incubated for 4 days at room temperature. Colonies were counted from three biological replicates, and mean and SD were calculated.

### FACS

10^6^ cells from the high- and low-expressing tails (top and bottom 10% of cells) of the HSE-YFP/side scatter distribution in wild-type, *hsp82*Δ, and *hsc82*Δ cells were sorted in the Whitehead Institute FACS facility on a BD FACS Aria.

### Estradiol Dose Responses

Estradiol dose responses were performed as described (Zheng et al., 2016).

## Supplementary Material

1

2

## Highlights

- Differential Hsf1 phosphorylation generates cell-to-cell variation
- Heterogeneous Hsf1 activity generates variation in the levels of the Hsp90 chaperone
- Variation in Hsp90 expression enables yeast cells to acquire novel phenotypes
- Hsf1 phosphorylation serves as a phenotypic capacitor by tuning proteostasis
