# Supplementary material for: Hsf1 Phosphorylation Generates Cell-to-Cell Variation in Hsp90 Levels and Promotes Phenotypic Plasticity
Source: Cell Rep. Author manuscript; Available in PMC 2018 Apr 10. (PMC5893160; doi:10.1016/j.celrep.2018.02.083)
Supplement: 1 [file NIHMS955386-supplement-1.pdf]

Cell Reports, Volume *22*

Supplemental Information

Hsf1 Phosphorylation Generates Cell-to-Cell Variation in Hsp90 Levels and Promotes Phenotypic Plasticity

Xu Zheng, Ali Beyzavi, Joanna Krakowiak, Nikit Patel, Ahmad S. Khalil, and David Pincus

Figure S1

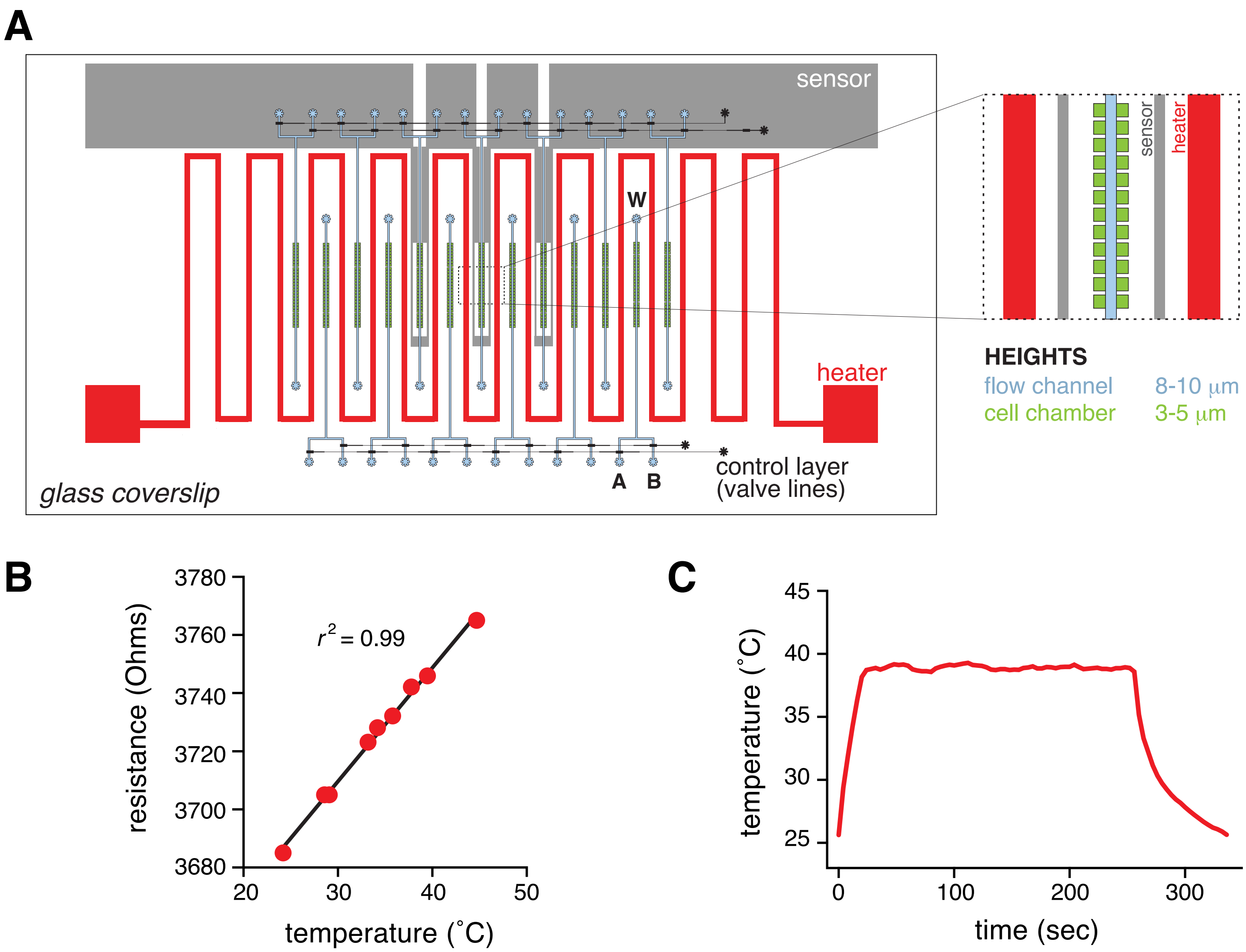

Figure S1. Characterization of the microfluidic heat shock device, related to Figure 2.
(A) Schematic of the microfluidic heat shock device. The assembled device consists of a glass coverslip patterned with Pt/Ti heater and sensor wires (grey), and a multilayer PDMS device with flow (black and blue channels) and control (red channels) layers. Cells loaded from either port A or B are trapped in chambers (blue) that have been fabricated to the height of a single monolayer of S. cerevisiae cells. W denotes waste port.
(B) Representative calibration curve for the on-chip sensor showing a linear response across the operating temperature range.
(C) Using the on-chip heater to achieve and accurately maintain chamber temperature of 39˚C, as measured by a high-resolution long-wave infrared thermal camera.

Figure S2

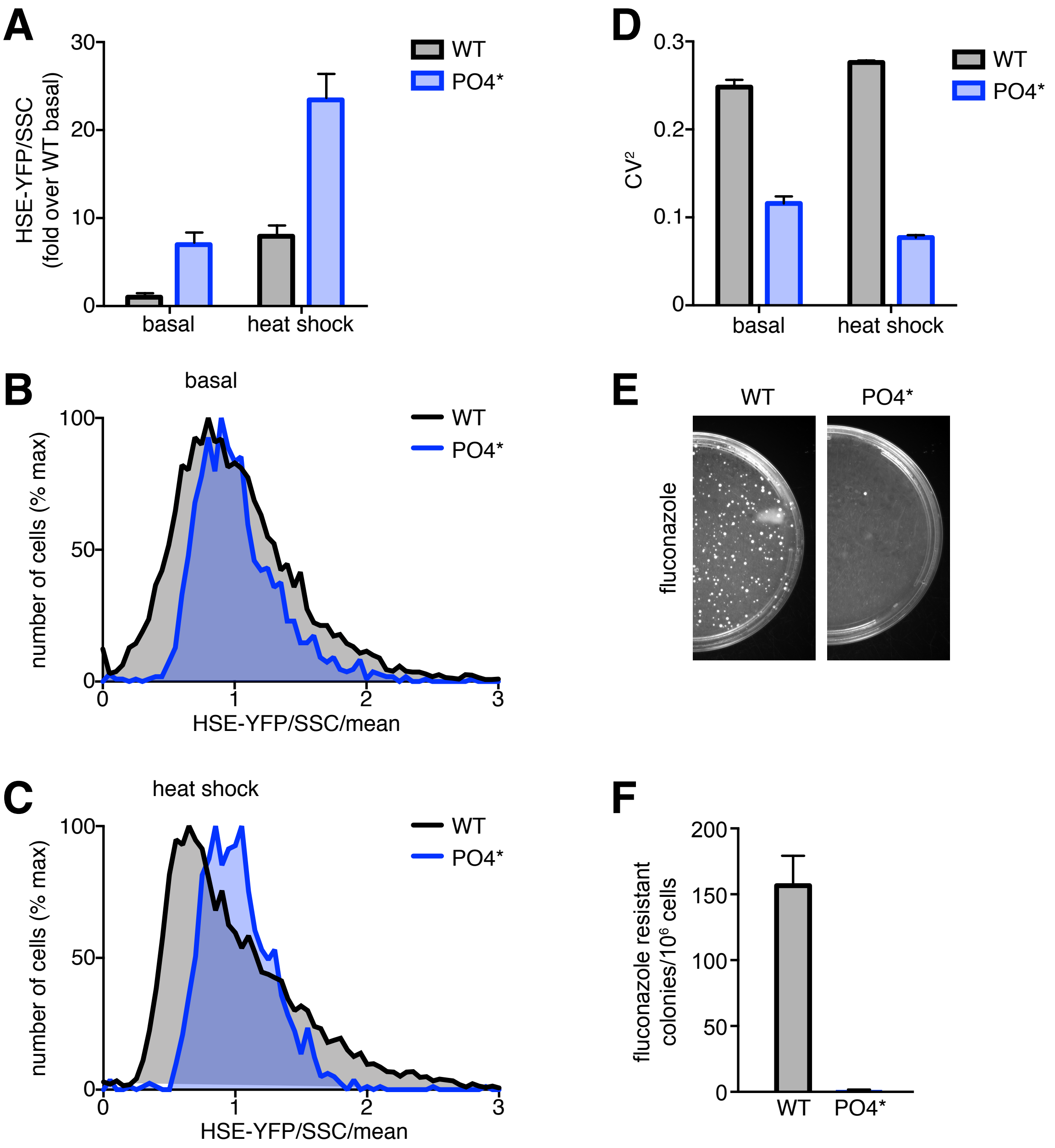

Figure S2. Hsf1PO4* shows reduced cell-to-cell variation and fluconazole resistance, related to Figure 3.

(A) Cells expressing either wild type Hsf1 or the 116 aspartate substitution phospho-mimetic Hsf1-PO4* were measured for Hsf1 activity using the HSE-YFP reporter under basal and heat shock conditions (4 hrs at 39ºC). The average of the median of the HSE-YFP/SSC distribution from 3 biological replicates is plotted, and the error bars are the standard deviation from the replicates.

(B) Representative HSE-YFP distributions from wild type and Hsf1PO4* cells under basal conditions are overlaid following normalization by side scatter and the mean of the distribution.

(C) As in (B) but under heat shock conditions.

(D) Cell-to-cell variation in Hsf1 activity as measured by the CV2 of the HSE-YFP/SSC distributions are shown for wild type and Hsf1PO4* cells under basal and heat shock conditions. Error bars are the standard deviation of 3 biological replicates.

(E) Appearance of fluconazole-resistant colonies in wild type cells and Hsf1PO4* cells. 106 cells were plated on YPD plates supplemented with 128 µg/ml fluconazole and incubated at room temperature for 4 days.

(F) Quantification of the number of fluconazole-resistant colonies in wild type and Hsf1PO4* cells.

Figure S3

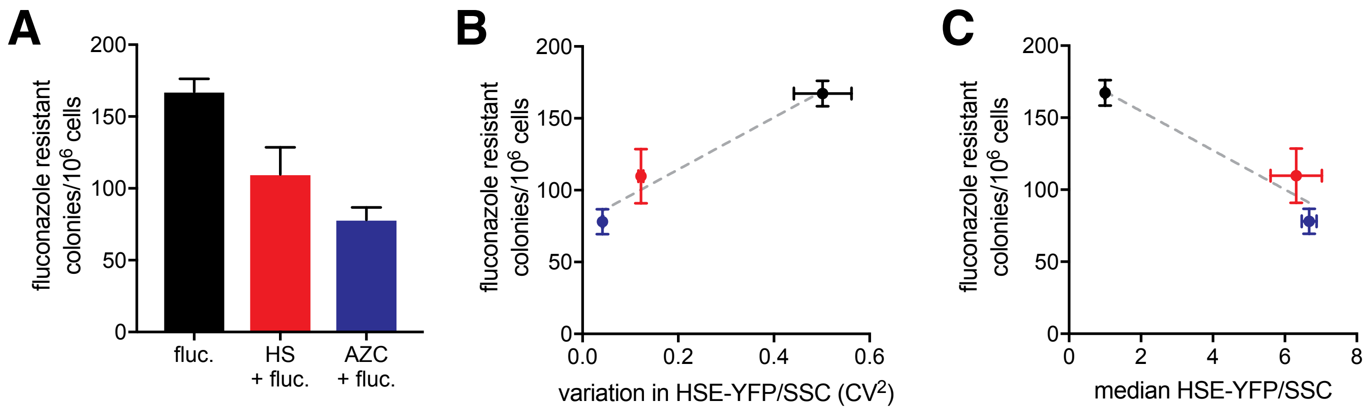

Figure S3. Acquired fluconazole resistance in cells pretreated with heat shock or AZC, related to Figure 4.

(A) Quantification of the number of fluconazole-resistant colonies in wild type cells that were either untreated, heat shocked at 39ºC for 4 hours or treated with 4 mM AZC for 4 hours prior to plating on fluconazole plates. Error bars are the standard deviation of 3 biological replicates.

(B) Fluconazole resistance positively correlates with cell-to-cell variation in Hsf1 activity as measured by the CV^2 of the HSE-YFP/SSC distribution. The CV^2 data are from the untreated samples Figure 1. Black is untreated, red is heat shocked, blue is AZC treated.

(C) Fluconazole resistance negatively correlates with absolute Hsf1 activity as measured by the median of the HSE-YFP/SSC distribution. Median data is from Figure 1. Black is untreated, red is heat shocked, blue is AZC treated.

Table S1. Results Levene's test of variances, related to all figures.

Figure 1: HS vs AZC (HSE-YFP/SSC)

time (hrs)	p-value
0	0.92
0.5	< 0.01
1	< 0.01
1.5	< 0.01
2	< 0.01
2.5	< 0.01
3	< 0.05
3.5	< 0.05
4	< 0.05

Figure 2A: WT vs Δpo4 (HSE-YFP/SSC)

time (min)	p-value
0	< 0.01
10	< 0.01
20	< 0.05
30	< 0.01
45	< 0.01
60	< 0.05
90	< 0.05
120	< 0.05

Figure 3B: Hsp82-YFP/SSC (WT vs Δpo4)

p-value	0.08

Figure 4E: WT vs Δpo4 (HSE-YFP/SSC)

[estradiol]	p-value
1	< 0.05
8	< 0.05
32	< 0.01

Table S2. Yeast strains used in this study, related to all figures.

DPY number	Description	Genotype
144	WT HSE-YFP	W303 MATa 4xHSE-Venus::LEU
118	Hsf1-3xFLAG/V5	W303 MATa *hsf1*Δ::KAN Hsf1-3xFLAG-V5::TRP1
416	Hsf1Δpo4 HSE-YFP	W303 MATa *hsf1*Δ::KAN 4xHSE-Venus::LEU2 Hsf1Δpo4::TRP1
605	Hsf1PO4* HSE-YFP	W303 MATa *hsf1*Δ::KAN 4xHSE-Venus::LEU2 Hsf1PO4*::TRP1
1221	hsc82Δ HSE-YFP	W303 MATa 4xHSE-Venus::LEU2 *hsc82*Δ::HYG
1222	hsp82Δ HSE-YFP	W303 MATa 4xHSE-Venus::LEU2 *hsp82*Δ::HYG

SUPPEMENTARY METHODS

Microfluidic heat shock time courses

Single cell heat shock experiments were performed with a custom microfluidic device that uses microscale, on-chip heaters to enable programmable thermal perturbations. The multilayer device was fabricated out of the silicone elastomer polydimethylsiloxane (PDMS/Sylgard 184, Dow Corning) using soft lithographic techniques, as described previously (Duffy et al., 1998; Thorsen et al., 2002; Unger et al., 2000; Vega et al., 2012). The device was aligned and sealed to a pre-cleaned No. 1.5 glass coverslip (Fisher Scientific), onto which micro-scale heater and resistance temperature detector (RTD) wires were patterned. Briefly, a thin layer of Ti (100 Angstroms) was deposited on the glass, followed by a layer of Pt (150 nm) and finally a layer of SPR 220-7 positive photoresist (MicroChem Corp.). After transferring the heater/RTD pattern to the photoresist using a high-resolution transparency photomask (CAD/Art Services, Inc.), the Pt/Ti layers were etched in Aqua Regia solution to generate the final serpentine pattern of heater and RTD wires. The photoresist was then washed off with acetone, and a layer of SiO_2 (300 nm) was deposited on the final pattern. After assembly, the RTD of each device was calibrated and confirmed to respond linearly to changes in the input temperature, as controlled by a hot plate, across the operating temperature range of 25˚C to 45˚C (Figure S1). Additionally, the devices were characterized by applying user-defined voltages to the heater and measuring on-chip temperatures using a Flir A655sc high-resolution long-wave infrared thermal camera (Flir Systems, Inc.) to validate the device’s ability to achieve accurate and stable heat shock conditions (Figure S1).

To perform microfluidic experiments, we used a custom microfluidic platform that controls the delivery of liquids to the device and the actuation of valves (Vega et al., 2012). The voltage across the heater wire and the resistance across the RTD wire were controlled and measured, respectively, to modulate the on-chip temperature. For each experiment, cells were inoculated

1:100 from overnight SDC cultures into 2 mL SDC and allowed to grow 3-4 hours (OD ~0.5) before seeding the device. Cells were then loaded into the device to trap a small number of cells in growth chambers. Cells were grown on-chip in SDC media for 4-5 hours at 25˚C, ambient conditions maintained with a Controlled Environment Microscope Incubator (Nikon Instruments, Inc.) designed for live-cell imaging. Cells were then subjected to a heat shock by applying voltage across the heater wire to ramp to and maintain an on-chip temperature of 39˚C.

Throughout, images were collected at 15-minute intervals at 100x magnification (Plan Apo Lambda 100X, NA 1.45) using an Eclipse Ti-E inverted microscope (Nikon Instruments, Inc.) equipped with the “Perfect Focus System”, a XYZ-motorized stage, and a Clara-E charge-coupled device (CCD) camera (Andor Technology). Images were acquired in phase contrast configuration and in the YFP fluorescent channel. Filters and light sources (Nikon LED and Lumencor SPECTRA X Light Engine) were automatically controlled using the supplier’s software (NIS-Elements Advanced Research). Following each experiment, cells were segmented using custom image analysis software written for Matlab (Mathworks, Natick, MA), and fluorescence values for each cell, averaged over cell area, were obtained from the time series of YFP images.
